# Supplementary material for: PlasmidMaker is a versatile, automated, and high throughput end-to-end platform for plasmid construction
Source: Nat Commun. 2022 May 16;13:2697. doi: 10.1038/s41467-022-30355-y (PMC9110713; doi:10.1038/s41467-022-30355-y)
Supplement: Supplementary file 1 — Supplementary Information [file 41467_2022_30355_MOESM1_ESM.docx]

Supplementary Information for

PlasmidMaker is a Versatile, Automated, and High Throughput End-to-End Platform for Plasmid Construction

*Enghiad et al.*

**This document includes:**

[Supplementary Text 2](#_Toc101990830)

[Optimization of ligation parameters for assembly of multiple linear DNA fragments 2](#_Toc101990831)

[Detailed design of guides and primers 2](#_Toc101990832)

[Description of different Python modules used for picklists generation and analysis 5](#_Toc101990833)

[Supplementary Figures 7](#_Toc101990834)

[Supplementary Tables 21](#_Toc101990835)

[Supplementary Data 26](#_Toc101990836)

[Supplementary References 27](#_Toc101990837)

# Supplementary Text

## Optimization of ligation parameters for assembly of multiple linear DNA fragments

Following determination of the versatility of *Pf*Ago/AREs in efficient cleavage of different DNA sequences, we sought to investigate the effect of sticky end size and choice of DNA ligase on DNA assembly fidelity and number of correct colonies for assembly of multiple DNA fragments. To this end, we designed a five-fragment assembly for a 7.2 kb plasmid (pAmp-ZeaX) harboring a functional zeaxanthin pathway in *E. coli* (**Supplementary Fig. 5a**) and used a set of PCR primers and DNA guides creating either 9 or 12 nt sticky ends. Each of the five fragments was PCR-amplified and the mixture of fragments was digested in a one-pot reaction using *Pf*Ago/AREs. The purified digestion products were then assembled by T4, T7, or *E. coli* DNA ligase and the DNA assembly fidelity and the number of correct colonies for each assembly were calculated. As shown in **Supplementary Fig. 5b**, we were able to acquire correct assembly products for all the sticky end sizes and DNA ligases tested. Between the DNA ligases, *E. coli* DNA ligase demonstrated the highest assembly fidelity (>95%) as well as total number of correct colonies (>9,000) for both 9 and 12 nt sticky ends. No significant difference in assembly fidelity and number of correct colonies between 9 and 12 nt sticky ends was observed. As 12 nt AREs provide higher cleavage specificities, we decided to continue our optimizations on 12 nt sticky ends.

To investigate the effect of ligation temperature, we designed two new sets of DNA guides which create different 12 nt sticky ends compared to the previous set and examined the effect of ligation temperature on DNA assembly for the three sets. The digested products for each set were incubated with *E. coli* DNA ligase for 2 hours at 25, 37, or 45 °C as well as a condition in which the ligation temperature was shifted between 37 and 25 °C for 30 seconds periods and a total of 2 hours. As shown in **Supplementary Fig. 5c**, for the three tested sets, ligation temperature did not have any major effect on DNA assembly fidelity and total number of correct colonies. As a result, for 12 nt sticky ends, DNA ligation can be performed at a flexible temperature range of between 25 and 45 °C.

In our previous assemblies, *E. coli* DNA ligase demonstrated the highest assembly fidelity. However, DNA ligation at elevated temperatures (i.e. higher than 37 °C) allows the use of DNA ligases from thermophilic organisms for assembly of DNA molecules, which can potentially offer higher ligation specificities^1-3^. Compared to T4 and *E. coli* DNA ligases, DNA ligase from *Thermus thermophilus* (i.e. *Taq* DNA ligase) exhibits substantially higher discrimination against base pair mismatches^4^. Therefore, we decided to test the specificity of *Taq* DNA ligase and a commercially available engineered version of this enzyme, HiFi *Taq* DNA ligase (New England Biolabs, MA), in ligation of mismatched 12 nt sticky ends. For this purpose, we generated two fragments with 12 nt mismatched sticky ends by *Pf*Ago/AREs and performed DNA ligation between the two fragments using *E. coli*, *Taq* or HiFi *Taq* DNA ligase. The generated sticky ends shared seven identical nucleotides at one end, and one identical nucleotide at the other end. As shown in **Supplementary Fig. 5d**, incubation of the two fragments using *E. coli* or *Taq* DNA ligase resulted in creation of a mismatched ligation product. However, interestingly, HiFi *Taq* DNA ligase was able to discriminate between the two mismatched sticky ends as no visible ligation product was observed. Therefore, HiFi *Taq* DNA ligase offers higher assembly fidelities compared to both *E. coli* and *Taq* DNA ligases and was selected as the choice of ligase for assembly of DNA fragments generated by 12 nt *Pf*Ago/AREs.

## Detailed design of guides and primers

To generate guides and primers for the plasmid construction, we first obtain the plasmid sequence by concatenating the nucleotide sequence in the specified order of assembly. For the assembly of ‘n’ fragments, we search for the optimal 24 bp recognition sequence at each junction of assembly to generate 12 bp sticky ends. The 24 bp recognition sequence contains 2 guides of 16 bp each. The guide search space is generated by concatenating 40 bp each from the end of the adjoining fragments and then, a guide library is constructed by acquiring all possible 24 bp nucleotide sequences out of the guide search space. Similarly, we construct an off-target library by extracting 22 bp from both strands of the plasmid sequence. For off-target comparison, the nucleotides at the end are not considered. Once all the libraries are created, we start searching through the guide library to find suitable guides which satisfy intra-fragment and inter-fragment criteria. These rules are designed based on the experience.

We calculate the GC-content of the fragments and the guide search space to suggest the user whether to use WT *Pf*Ago + *Pf*Ago* or *Pf*Ago* alone for the plasmid construction. If more than half of the fragments have GC-content greater than 57% and if any junction for searching guides, i.e., guide search space has GC-content greater than 65%, we recommend using only the *Pf*Ago*. To find the suitable guides for creating desired sticky ends, we go through the list of 57 possible recognition sequences for each junction. Recognition sequences are first selected based on the intra-fragment criteria. Selections of *Pf*Ago enzyme and recognition sequences GC-content were guided by the data in **Fig. 2** and **Supplementary Figs. 4a-c**.

The intra-fragment criteria consist of the following:

1. GC-content:

24 bp recognition sequence contains two guides which bind to each DNA strand.

If using WT *Pf*Ago + *Pf*Ago*,

- The GC-content of the recognition sequence excluding the nucleotide at the end (22 bp) should be between 15% to 60%
- For individual guides excluding the first and last nucleotides (14 nt), the GC-content should be between 15% to 65%

If using *Pf*Ago*,

- The GC-content of the recognition sequence excluding the nucleotide at the end (22 bp) should be between 30% to 75%
- For individual guides excluding the first and last nucleotides (14 nt), the GC-content should be between 15% to 65%

Higher priority is given to the guides with lower GC-content for higher cleavage efficiency. Therefore, we start with different upper limit constraints on GC-content starting from 45% with an increment of 5% for the recognition sequence till 60% and from 50% with an increment of 5% for the individual guides if we are using the WT *Pf*Ago + *Pf*Ago*. If we are only using the *Pf*Ago*, all the GC-content constraint values are increased by 5%.

1. Presence of G-quadraplex and sequences containing 5 or more consecutive A’s or T’s

Recognition sequence is rejected if 22 bp region contains either

- G-quadraplex on either strand or
- At least 5 consecutive A or Ts

1. Off-target scores

To ensure that *Pf*Ago has negligible off-target activity, we compare the 22 bp of the recognition sequence with the off-target library based on sequence similarity. We define two scores, namely –

- 1. Guide Homology Score

Discard the guides if recognition sequence homology >= 15 nucleotides (default)

- 1. 3 Fragment Homology Score

Discard the recognition sequence if sequence homology in 22 bp and middle 6 bp region is greater than or equal to 13 and 5 respectively (default)

1. Self-Ligation

Recognition sequence is discarded if the sticky end generated is palindromic. Once we find the recognition sequences satisfying intra-fragment criteria for all the junctions, sticky ends are generated to check for potential mismatch ligation. We use the terms - mismatch ligation check and inter-fragment criterion interchangeably. We define the mismatch ligation score (MLS) using a quadratic penalty. The penalty decreases as we move towards the center of 12 bp sticky end. G-T mismatch ligation is also accounted with a weight of 0.75. A matrix is created to obtain MLS for all combination of mismatch ligation between sticky ends. If one of the guides do not satisfy the inter-fragment criterion (MLS < 100), the algorithm looks for a replacement by considering other recognition sequences in the guide library for the respective junction. We keep doing this till all the mismatch ligation scores in the matrix are less than 100.

Depending on the plasmid size, GC-content and other factors, one might not be able to find the guides using the defined parameters. Therefore, to ensure guides are found for the assembly, we search the parameter by decreasing the stringency level. For example, if we do not obtain the guides using the rule of ‘Presence of G-quadraplex and sequences containing 5 or more consecutive A’s or T’s’, we decrease the stringency and only reject the guides with

- G-quadraplex on either strand or
- At least 6 consecutive A or T’s

Similarly for the off target, the code starts at the homology score parameters of [15,13,5] and the guides are searched till the parameters of [17,16,6] to find the guide.

Once the guides are obtained, primers required for creating amplicons with overhangs are designed as per the position of the recognition sequence. We initially select an 18 bp primer binding site and determine its GC-content. Depending on the GC-content, we decide the initial length of the primer binding site to start designing primer (**Supplementary Fig. 9**). The T_m_ values of the forward and reverse primers are calculated using ‘calcTm’ function of Primer3^5^. We increase the size of the primer binding region with lower T_m_ until T_m_ of the forward and reverse primers have a difference less than 2^o^ C. All the annealing temperatures are calculated from the NEB website.

In order to make the assembly work even for smaller fragments (< 80 bp), we use an updated workflow. Fragments smaller than 80 bp are first divided into 2 halves. Primers are designed so that these small fragments can be merged into adjacent fragments via overhangs in PCR amplification. We then obtain the guides and the primers for the modified fragments to perform one-pot scarless fragment assembly. Note that for the updated design, consecutive small fragments (<80 bp) need to be avoided.

For the algorithm to verify correct plasmid assembly, the list of the restriction enzymes shown **Supplementary Table 1** was used. This list can be modified by adding the necessary information in the ‘common_re_list.xlsx’ file depending on the restriction enzyme on hand. A stand-alone script to generate the list of restriction enzymes for plasmid assembly verification is also provided on the GitHub repository and is summarized in **Supplementary Fig. 10**. To use the script, the user is required to annotate the fragments as ‘fragment X’ or ‘Fragment X’ as shown in **Supplementary Fig. 8**.

We also addressed multiple problems observed during the plasmid construction using quality control scripts. One potential problem observed during the assembly of the plasmid library was presence of a repeats inside a single fragment which overlapped with the primer binding site. While performing the PCR reactions, this resulted in smaller PCR fragments. We resolve this issue by adding a step in the quality control script where we search for potential repeats (>15 bp) in all the fragments individually and check whether one of the repeats is present in the potential primer binding region. Another problem we faced was high junction similarity in the case of *I. orientalis* plasmids. Therefore, once the order for plasmid is submitted, we also check the assembly junctions for sequence similarity. If the similarity between any two junctions is greater than 75%, we provide a warning to the users regarding the potential difficulty in designing the guides. One limitation of the script is adding consecutive small fragments. We recommend the users to redesign the fragments such that the final plasmid map does not contain two consecutive fragments less than 80 bp. This can be resolved by merging two small fragments into a single fragment. Another quality control step checks for the same before the plasmid is submitted for guide and primer design.

## Description of different Python modules used for picklists generation and analysis

- Quality control I

This Python script checks for following problems in the annotated DNA sequence:

- - Duplicated/missing fragment annotations
  - Discontinuous DNA sequence between continuous fragments
  - Repeated DNA junctions in plasmid between fragments
  - Multiple primer binding sites in one fragment
  - Consecutive inserts of less than 80 bp
- Primer and guide design

This module is described in section “Detailed design of guides and primers”. The output of the Python script is a reference csv file for each plasmid that contains information about sequence name, sequence, T_m_, PCR product length, extension time and name of the PCR template.

- Quality control II

This module verifies that all primers and guides designed and present in the reference csv file for a plasmid are found in the DNA sequence of the plasmid.

- Primers and guides ordering

This Python module combines all primers and guides from all reference csv files and removes duplicated DNA sequences. It separates primers and guides and generates excel files for both 96-well plate and 384-well plate ordering consistent with the Integrated DNA Technologies (IDT) ordering format.

- Module 1

This Python module reads the information sheet from IDT and separates information of multiple plates into individual csv files. This module keeps the information about DNA sequence, sequence name, amount (nmoles), and well position from the original IDT information sheet and writes the individual csv files for each plate.

- Module 2

Using the edited information sheet from module 1, this Python module creates dilution picklists to add water to lyophilized DNA to make the concentration of 100 μM for 96-well plate and 200 μM for 384-well plate. Another picklist is generated that dilutes the primers 10-fold and transfers them to a Corning 384-well plate that will become the source plate for Echo acoustic liquid handler.

- Module 3

The primer and guide sequences from reference csv files are compared for exact sequence match with edited IDT information sheet from module 1 and the well position of primers and guides in the 96-well plate or 384-well plate are appended to the reference csv file as a separate column. A picklist is created to mix all guides for one plasmid into one well of a 96-well plate. Another picklist is created to add phosphorylation master mix to mixed guides.

- Module 4

This module creates the picklists for setting up PCRs. Using the modified reference csv file from module 3, it locates the position of forward and reverse PCR primers for each fragment and the name of the PCR template for that specific PCR fragment. The PCR template position is searched in the template position csv file. This Python module first creates a consolidated PCR picklist consisting of all PCRs required for one batch of assembly. The consolidated PCR picklist is divided into multiple PCR picklists if there are more than 92 PCRs and a position in the 96-well PCR plate is assigned to each PCR. When PCRs are divided into multiple PCR plates, it does not split the PCRs for one plasmid between multiple plates.

For extension PCR, a modified module 4 is used to generate extension PCR picklist first. The purified PCR fragments from extension PCR are transferred to an Echo source plate to be used as a template and their positions in the Echo source plate are added to the template positions csv file.

- Module 5

This module creates picklists for equimolar mixing of PCR fragments. It reads the output file from Lunatic (Unchained Labs, Pleasanton, CA) for DNA concentration of purified PCRs in the 96-well PCR plate. It creates a picklist for transfer of purified PCR fragments form a 96-well PCR plate to a 384-well plate that will be used as a source plate for mixing PCR fragments in Echo acoustic liquid handler. For each plasmid, the script calculates equimolar amount of PCR fragments with total DNA amount being 1 μg and creates a picklist for mixing of PCR fragments. It then creates a picklist for mixing the phosphorylated guides to the PCR fragment mix.

- Fragment Analyzer module

After the Fragment Analyzer run, the sizing data is exported in a csv file. This module compares the size of two most intense bands from a lane in the Fragment Analyzer (Agilent Technologies, Santa Clara, CA) against the expected PCR size from a reference csv file. The output csv file lists the results as correct or incorrect based on this comparison.

# Supplementary Figures


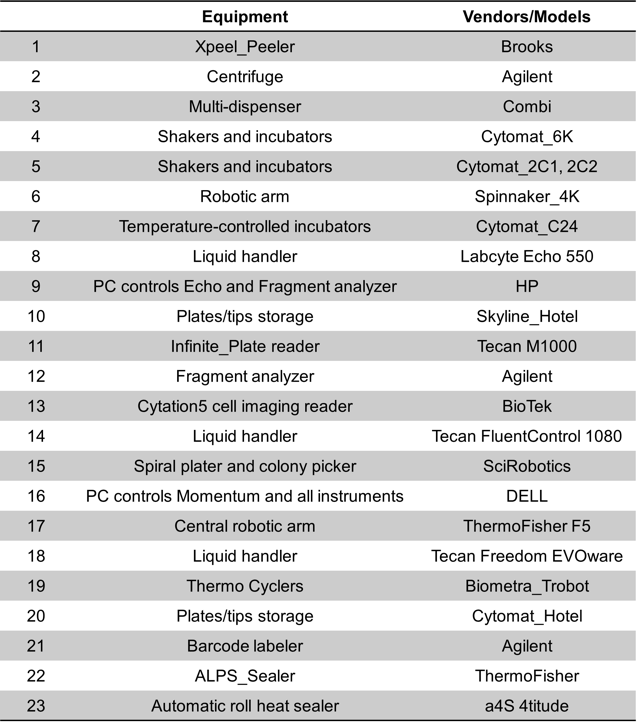

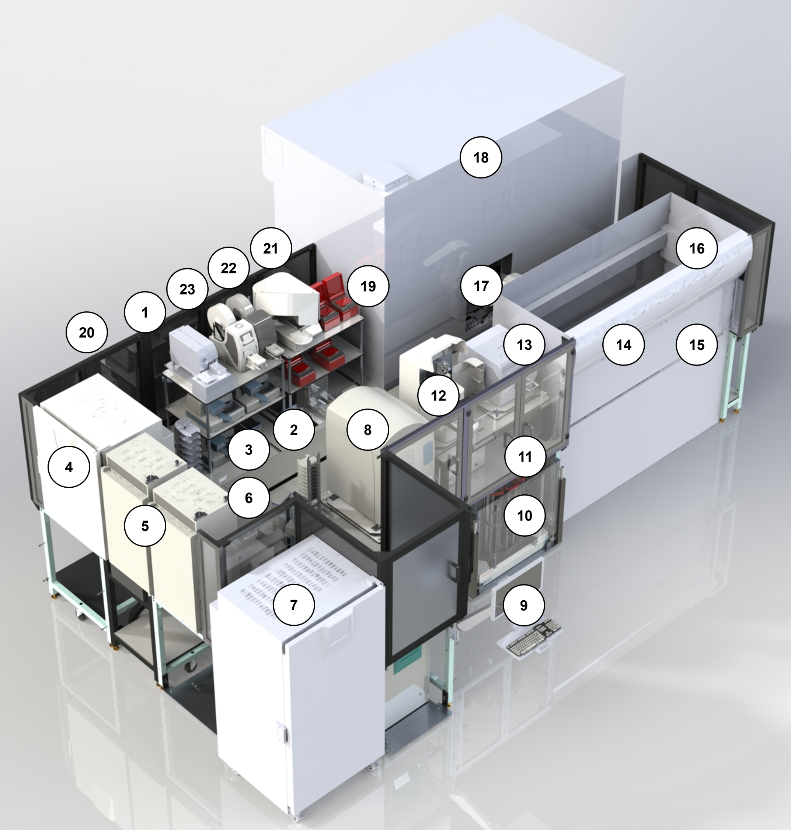


**Supplementary Fig. 1.** Overview of the iBioFAB system. Layout of the integrated hardware (#1-23) inside iBioFAB. A centralized 6-degree-of-freedom arm (#17_*ThermoFisher F5*) on a 6 m track is used to transport labware among devices.


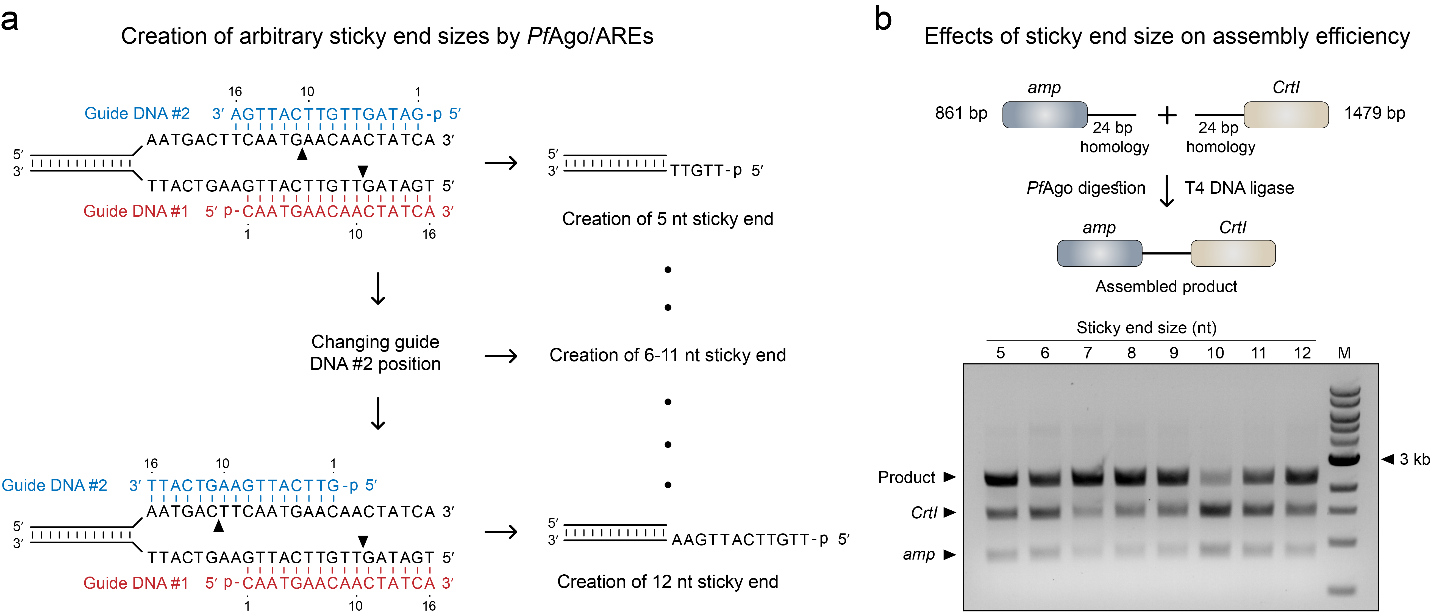


**Supplementary Fig. 2.** Characterization of *Pf*Ago/AREs capabilities in creation of user defined sticky ends of different sizes on linear DNA ends. **a)** The strategy used for creation of varying sticky end sizes on DNA ends. One guide DNA was designed to target the lower strand of the dsDNA molecule and create a nick after its 10^th^ nucleotide position. This guide DNA was kept constant for creation of all sticky end sizes. The second guide DNA was designed to target the upper strand of the dsDNA. By changing the second guide’s position, sticky ends of varying sizes can be created. **b)** Characterization of *Pf*Ago/AREs cleavage efficiencies in creation of 5-12 nt sticky ends on linear dsDNA molecules ends. *amp* and *CrtI* genes were amplified by PCR to share 24 bp sequence homology at their ends. The homology sequence is shown in part **a**. The amplified fragments were digested by *Pf*Ago/AREs and ligated by T4 DNA ligase. The assembly product was then analyzed using agarose gel electrophoresis. Except for 10 nt sticky ends, all other sticky ends can be efficiently used for DNA assembly applications. This experiment was repeated once with similar results. M: 1 kb DNA ladder.


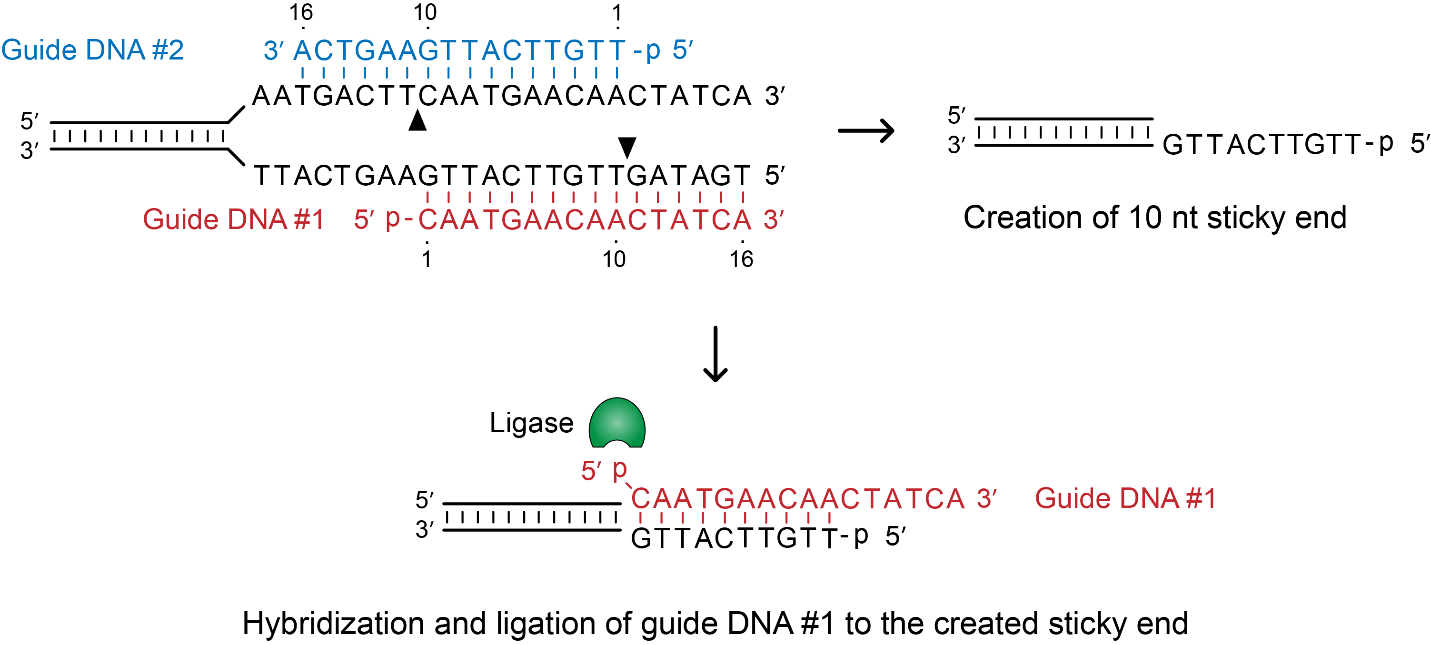


**Supplementary Fig. 3.** Interference of DNA guides with proper ligation of DNA fragments in case of 10 nt sticky ends. Because of complete homology of the first 10 nucleotides of guide DNA #1 to the created sticky end, this guide can also be ligated to the created sticky end and interfere with assembly.

**
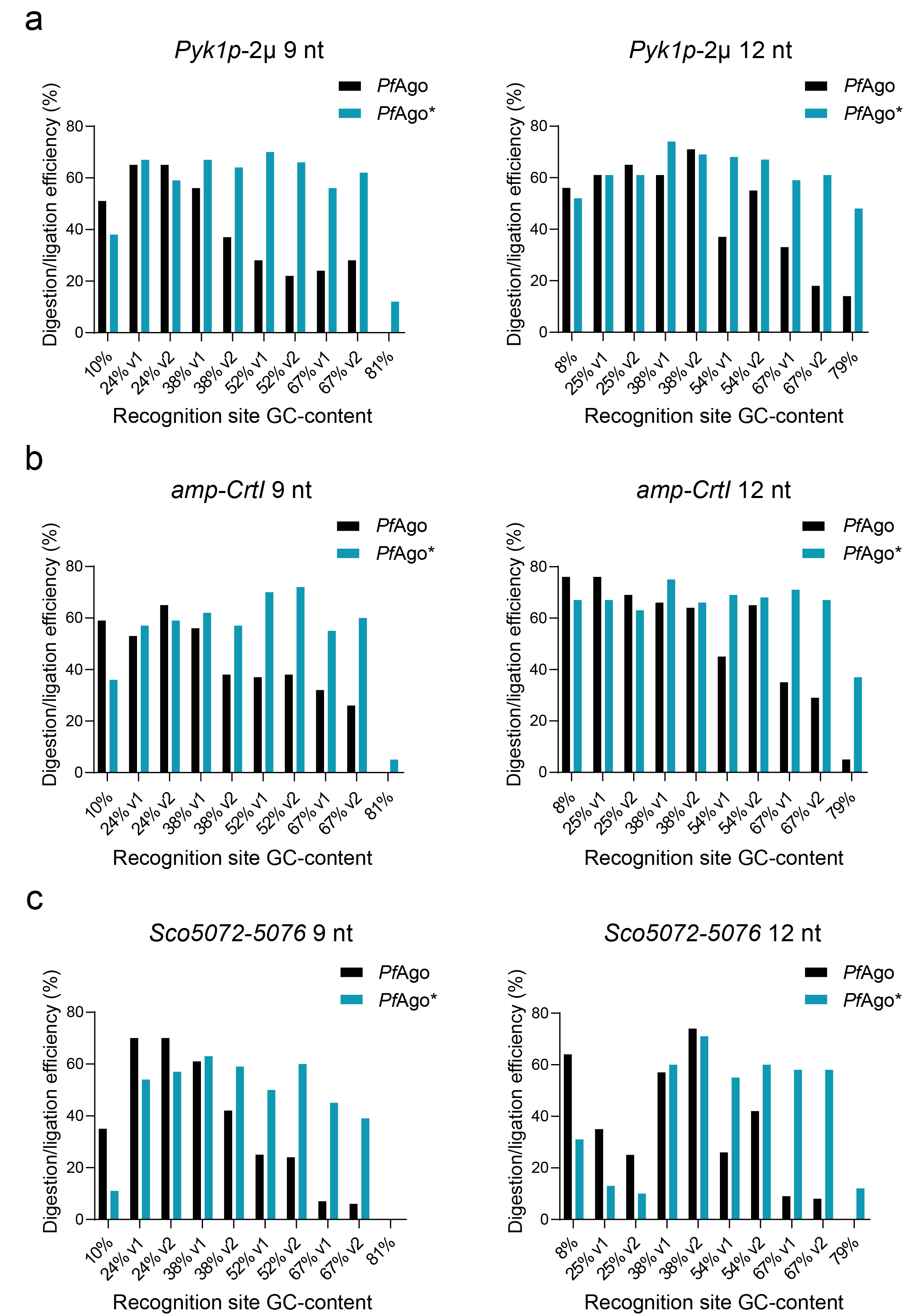
**

**Supplementary Fig. 4.** Analysis of the effects of *Pf*Ago/AREs recognition sequence GC-content and GC-distribution as well as the fragments overall GC-content on DNA assembly using *Pf*Ago/AREs. Cleavage/DNA assembly efficiency for WT *Pf*Ago and *Pf*Ago*/AREs creating 9 or 12 nt sticky ends on **a)** *Pyk1p*-2µ (~36% GC-content) **b)** *amp*-CrtI (~51% GC-content) and **c)** *Sco5072*-5076 (~73% GC-content) linear DNA sets. Source data are provided as a Source Data file. The assembly efficiency analysis for each set was performed only once.

**
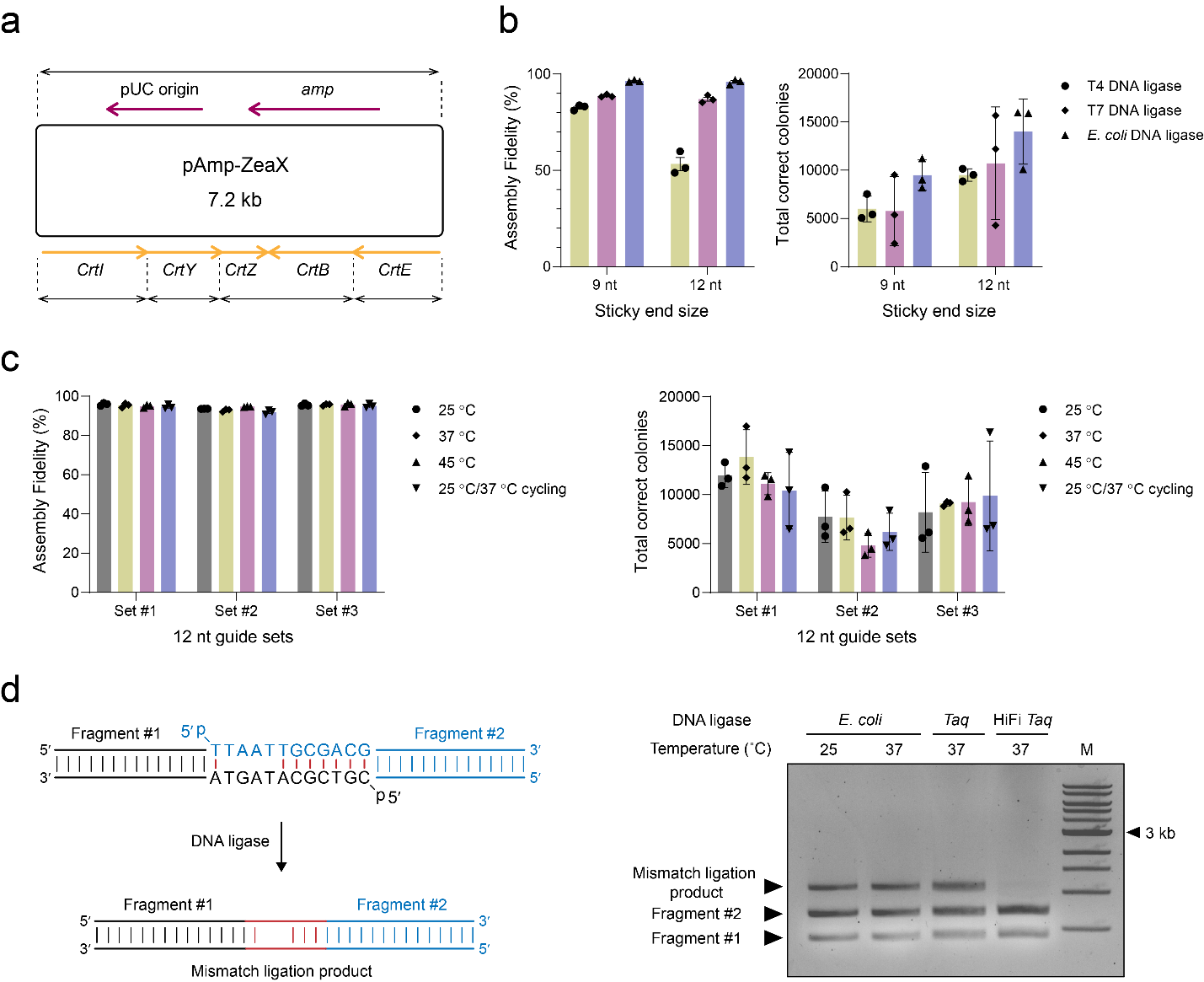
**

**Supplementary Fig. 5.** Optimization of ligation parameters for DNA assembly using *PfAgo*/AREs. **a)** DNA map for pAmp-ZeaX plasmid. The fragments used in the five-fragment assembly are shown by arrows. **b)** Effects of sticky ends size and choice of DNA ligase on assembly fidelity and number of correct colonies for the five-fragment pAmp-ZeaX assembly. Assembly fidelity was calculated based on the ratio of yellow colonies to total acquired colonies. All ligations were performed for 2 h at room temperature. All experiments were performed in three biological replicates. For total number of correct colonies and assembly fidelity, error bars show standard deviation (s.d.) and standard error (s.e.m) respectively. **c)** Effect of ligation temperature on assembly fidelity and number of correct colonies for three sets of five-fragment assemblies of a 7.2 kb plasmid. The *PfAgo*/AREs used for the experiments create 12 nt sticky ends. All experiments were performed in three biological replicates. For total number of correct colonies and assembly fidelity, error bars show standard deviation (s.d.) and standard error (s.e.m) respectively. **d)** Characterization of specificities of *E. coli*, *Taq*, and HiFi *Taq* DNA ligases in ligation of two mismatched 12 nt sticky ends. No visible mismatch ligation product was observed for HiFi *Taq* DNA ligase. M: 1 kb DNA ladder. This experiment was repeated one time with similar results.


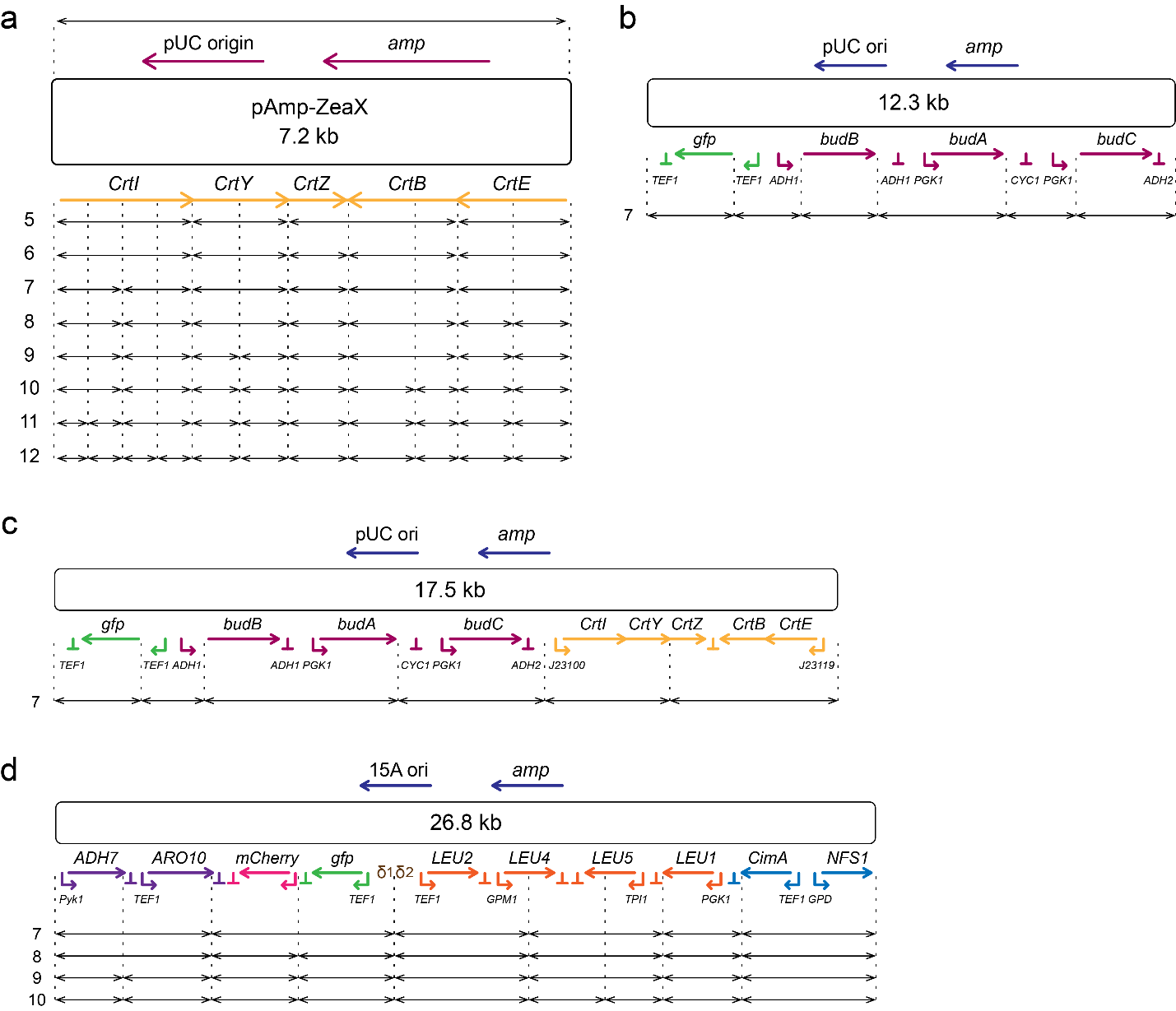


**Supplementary Fig. 6.** DNA maps for four plasmids used in characterization of method capabilities in terms of number of fragments and final product size. **a)** 7.2 kb plasmid harboring a functional zeaxanthin pathway in *E. coli*. **b)** 12.3 kb plasmid harboring an (*R,R*)-2,3-butanediol (BDO) pathway and a *gfp* gene. **c)** 17.5 kb plasmid harboring the BDO and zeaxanthin pathways and a *gfp* gene. **d)** 26.8 kb plasmid harboring an n-butanol pathway as well as *gfp* and *mCherry* genes. This plasmid contained 4 repeats of TEF1 promoter (~500 bp in size). The fragments used in each assembly are depicted as lines with arrows.


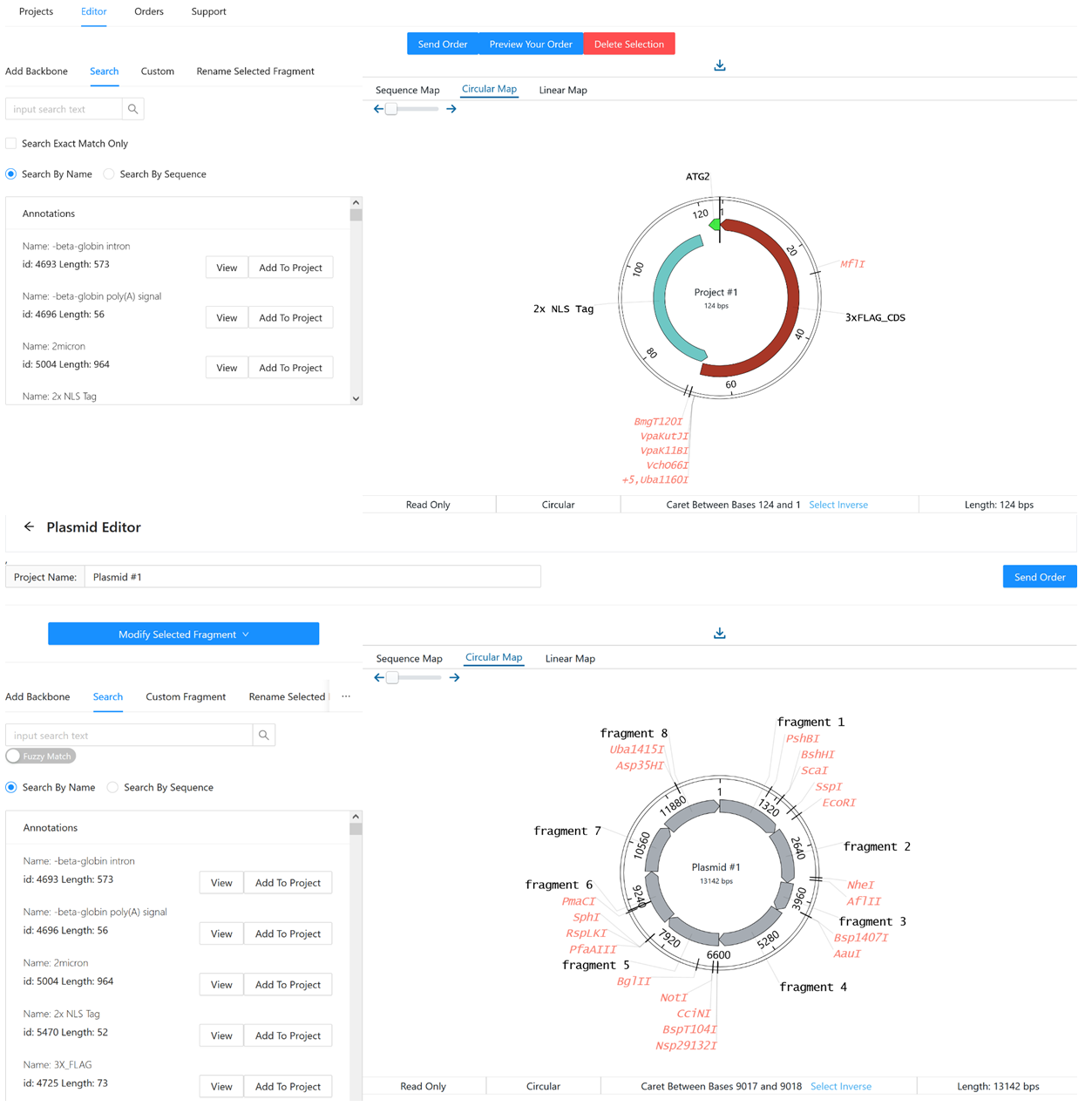


**Supplementary Fig. 7.** Project Editing Page. This page is where a user can build and edit their custom plasmid. On the bottom right is a visual status of the custom plasmid with all the annotations present whether they are promoters, genes, terminators, or other genetic parts. The bottom left of the page allows the user to search a particular genetic part for addition into the plasmid. The top left of the page allows the user to change the name of their project/order as well as change the ordering or direction of annotations in a custom plasmid. The top right of the page allows the user to submit the custom plasmid for potential production.

**
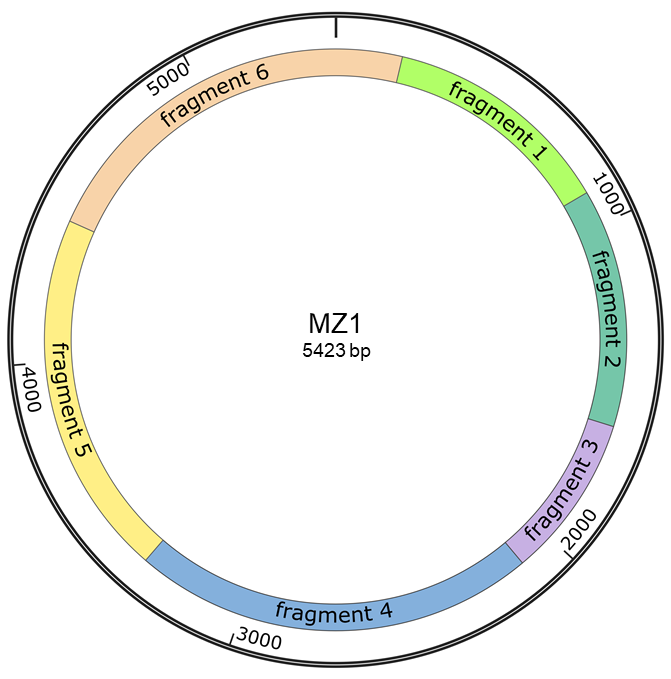
**

**Supplementary Fig. 8.** An example of annotated ‘.DNA’ plasmid map. The script to design guides and primers requires manual annotation of plasmid as ‘fragment X’ in the desired order of assembly. For a 6-fragments assembly of plasmid MZ1, individual fragments are annotated as ‘fragment 1’ to ‘fragment 6’. This map was generated using SnapGene viewer software.

**
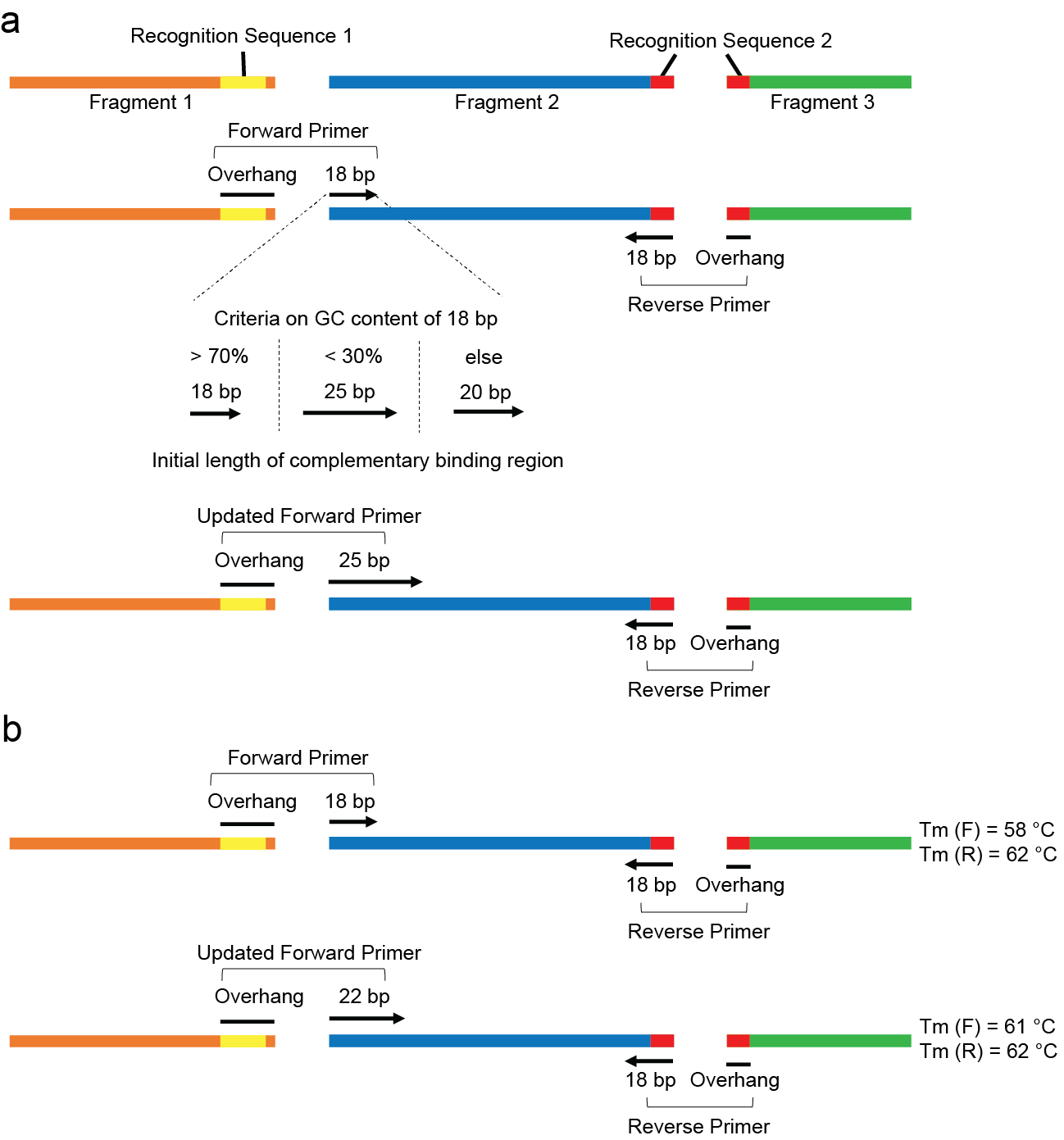
**

**Supplementary Fig. 9.** Primer design algorithm to amplify fragments. The primer consists of two parts – primer binding region and overhang. The overhangs are decided based on the recognition sequence obtained from the guide design algorithm. Determining the optimal primer binding region to amplify any fragment of interest is performed in 2 steps **a)** Decoding the initial length of primer binding site. Based on the GC-content for 18 bp of primer binding region, an initial length is selected to ensure working T_m_ values for PCR. **b)** Using Primer3^5^ to generate forward and reverse primers of similar T_m_ values. Once the initial lengths are fixed, the forward or the reverse primer are increased in length till the T_m_ value is within a range of 2 °C.

**
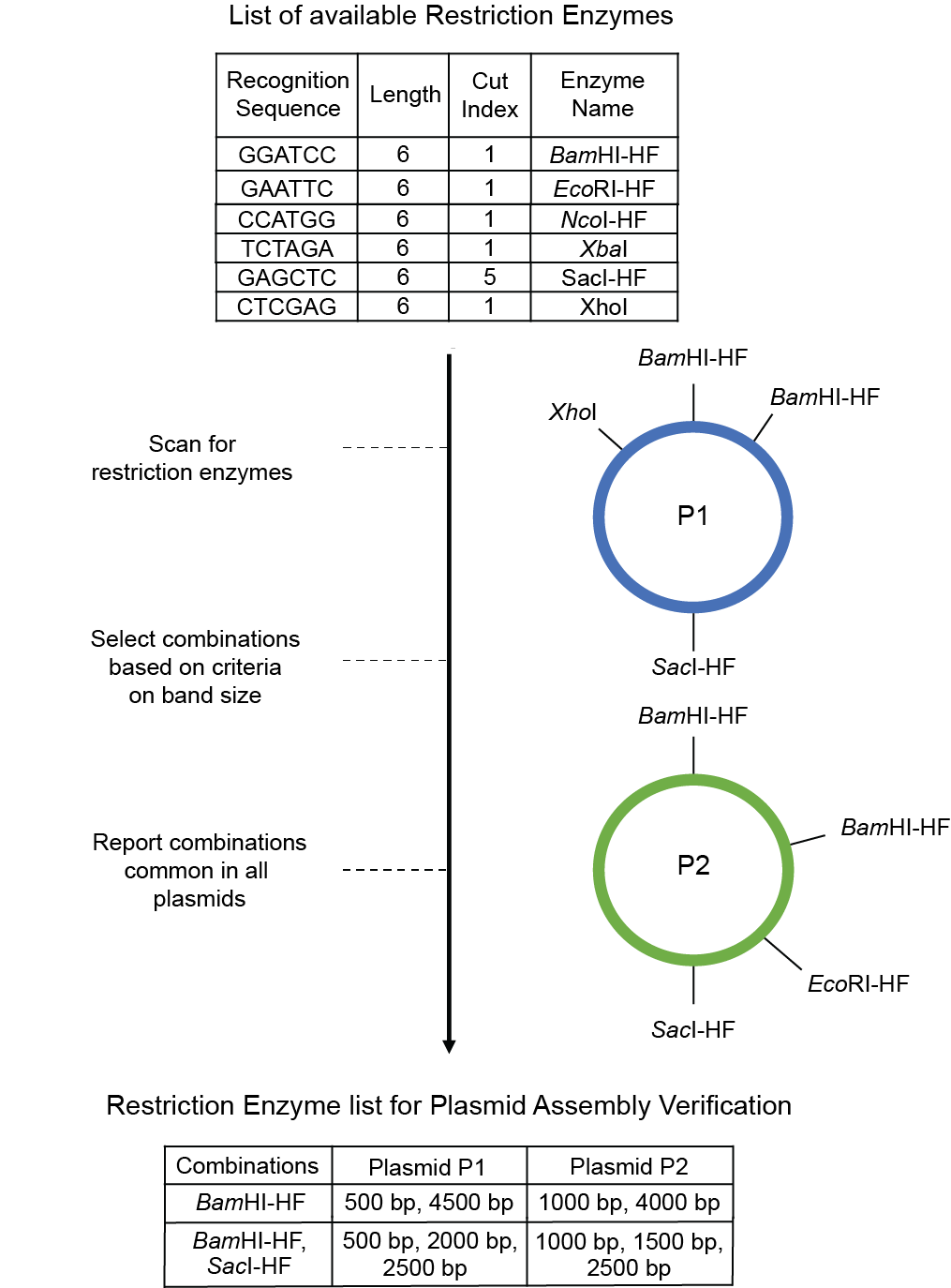
**

**Supplementary Fig. 10.** An example for selection of restriction enzymes to use in verification of assembled plasmids. In the example provided, the script scans the DNA sequence of plasmids P1 and P2 to find recognition sites for the restriction enzymes in the input list. The combinations of the enzyme are explored to generate observable pattern of bands during electrophoresis. The combinations commonly obtained across plasmids are reported as outputs for verifying constructed plasmids in a high throughput manner. In this case, the generated list will include 2 combinations for plasmid assembly verification of P1 and P2: 1. *Bam*HI-HF; 2. *Bam*HI-HF and *Sac*I-HF.


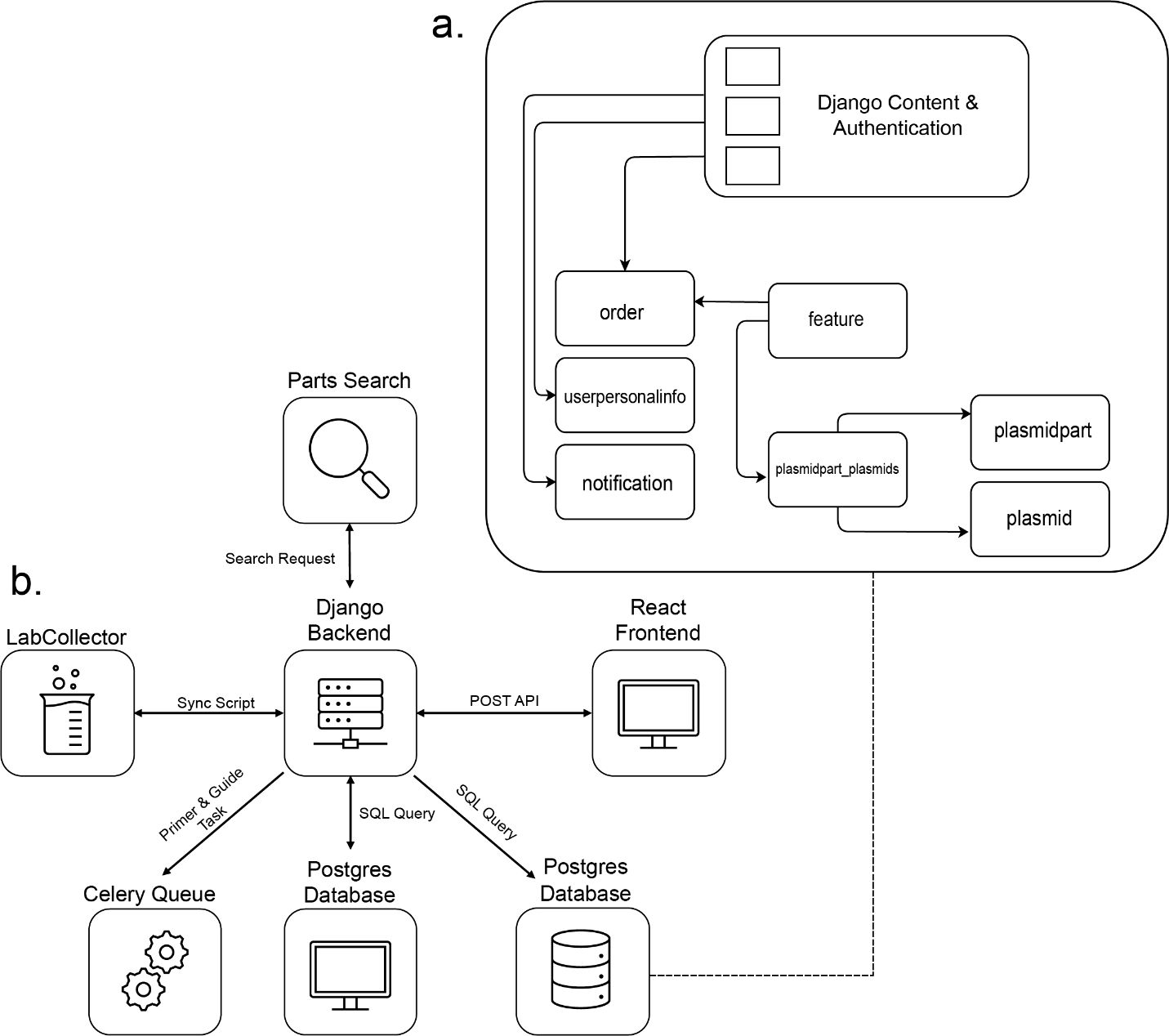


**Supplementary Fig. 11.** A detailed map of the interconnections between server components, consisting of **a)** A database diagram of how features and orders are stored; **b)** Communication between various components in the software stack.

**
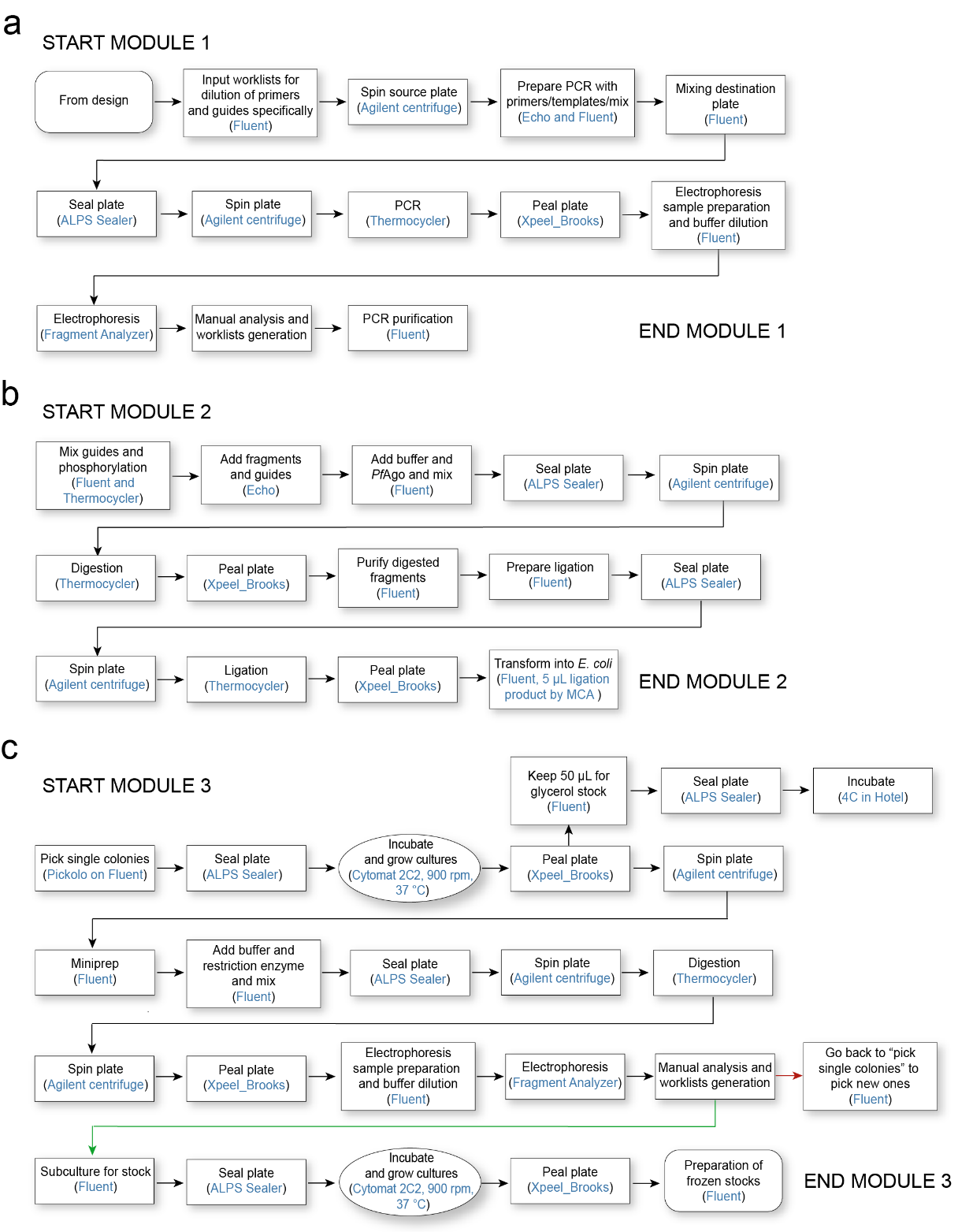
**

**Supplementary Fig. 12.** Detailed workflow of each Module of the automated plasmid construction pipeline including specific inputs, outputs, protocols being used, and potential human interventions for every single unit operation. **a)** Fragments preparation through PCR and purification. **b)** Automated assembly of fragments and transformation. **c)** Confirmation through plasmid extraction and gel electrophoresis. The red arrow means wrong digestion patterns on gel, and more colonies need to be picked for verification. The green arrow means correct digestion patterns on gel, and glycerol stocks can be made downstream. The steps of experiments are listed in black character and the corresponding instrument are listed in blue. Rectangle shape: automated steps without incubation and shaking. Oval shape: automated steps with incubation and shaking.

**
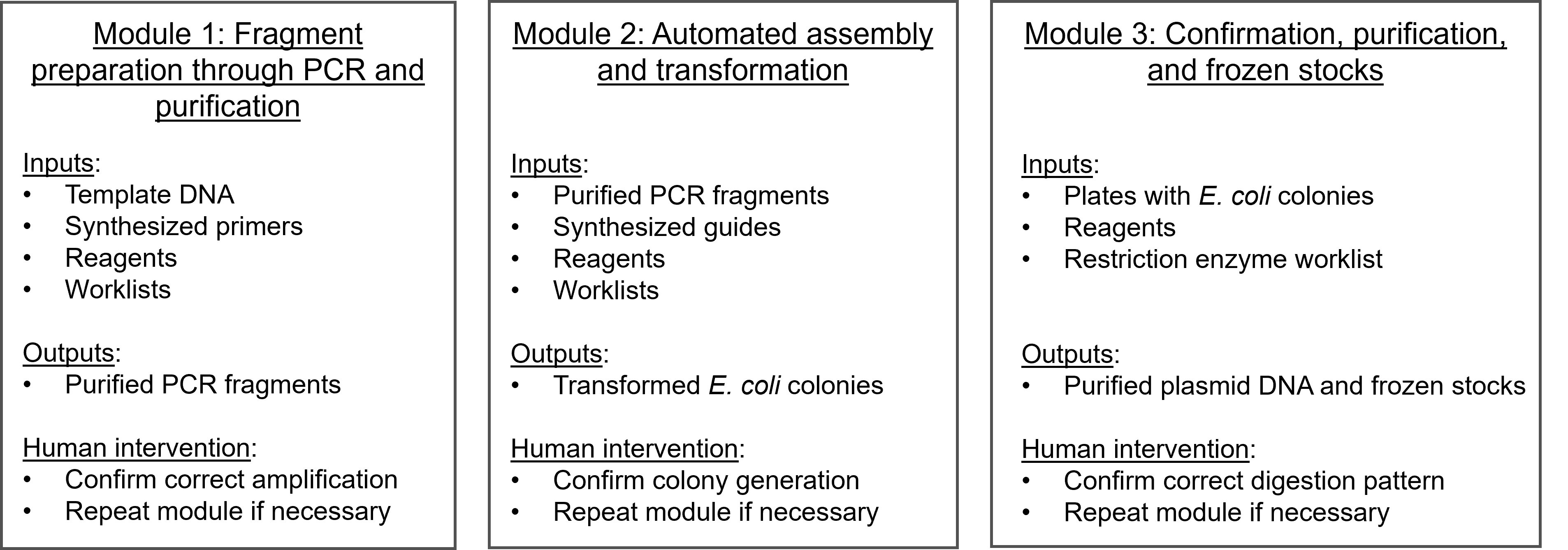
**

**Supplementary Fig. 13.** The inputs, outputs, and potential human intervention steps in each Module of the end-to-end automated plasmid construction workflow.

**
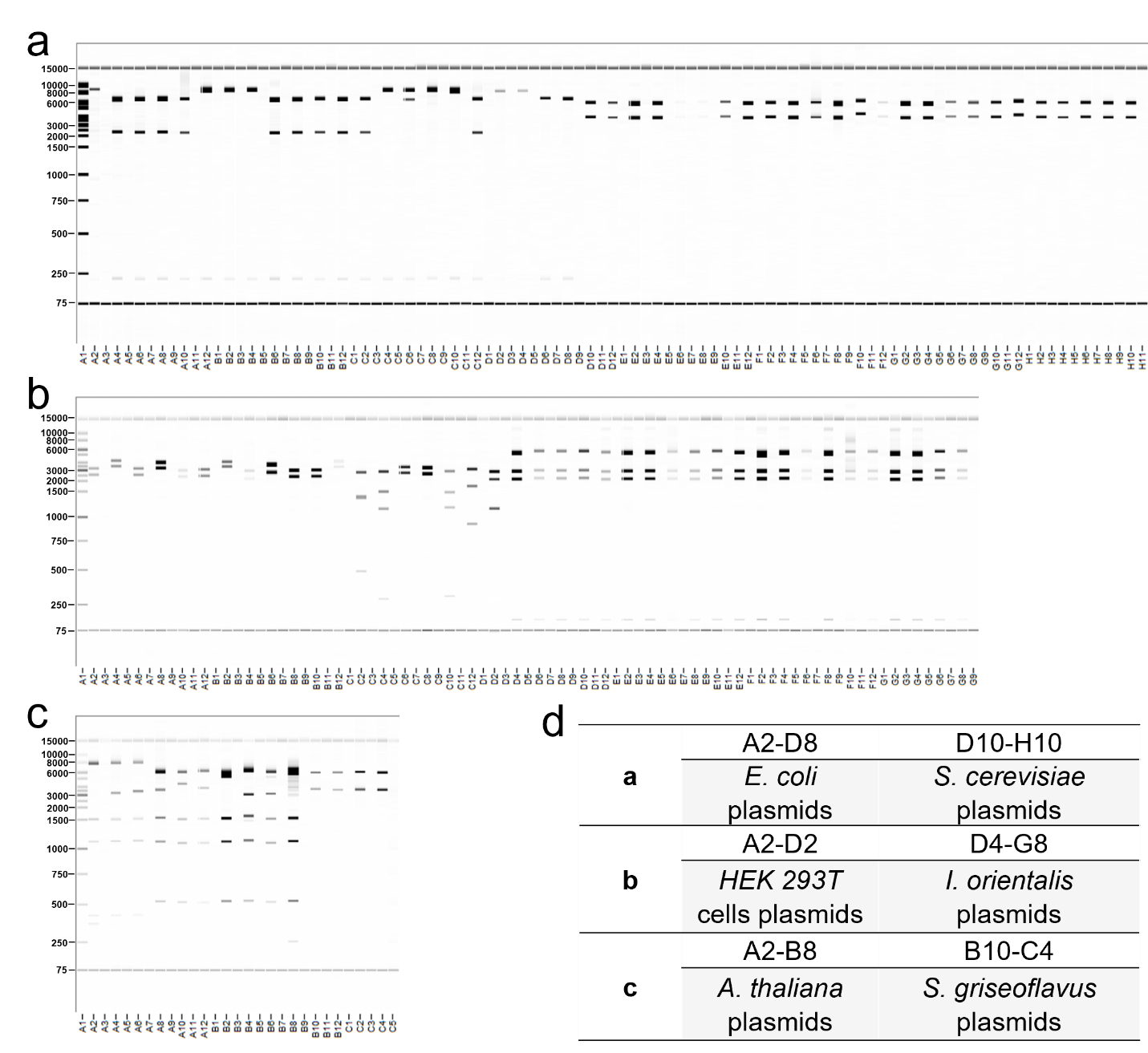
**

**Supplementary Fig. 14.** Verification of automated plasmid construction via double restriction digestion. The constructed 101 plasmids were checked by gel electrophoresis via Fragment Analyzer. The digestion patterns cover each of the constructed plasmids. The specific restriction enzymes being used for quality control in each category of plasmids are listed in **Supplementary Table 3**. All digestions showed correct band sizes according to the corresponding plasmid maps. **a)** Well A1 was 75-15,000 bp ladder (part number: DNF-920-K0500); Wells A2-D8 were plasmids for *E. coli*; wells D10-H10 were plasmids for *S. cerevisiae*; **b)** Well A1 was 75-15,000 bp ladder; wells A2-D2 wells were plasmids for *HEK 293T cells*; wells D4-G8 were plasmids for *I. orientalis*; **c)** Well A1 was 75-15,000 bp ladder; wells A2-B8 were *A. thaliana*; B10-C4 wells were *S. griseoflavus*; **d)** Legends for **a-c**. The automated construction of 101 plasmids were performed only once.

# Supplementary Tables

**Supplementary Table 1.** List of Commonly Used Restriction Enzymes**.**

| **Recognition Sequence** | **Length** | **Cut_index** | **Enyzme Name** |
| --- | --- | --- | --- |
| GGGCCC | 6 | 5 | *Apa*I-HF |
| GGATCC | 6 | 1 | *Bam*HI-HF |
| TGTACA | 6 | 1 | *Bsr*GI-HF |
| GAATTC | 6 | 1 | *Eco*RI-HF |
| GATATC | 6 | 3 | *Eco*RV-HF |
| AAGCTT | 6 | 1 | *Hind*III-HF |
| GTTAAC | 6 | 3 | *Hpa*I |
| GGTACC | 6 | 5 | *Kpn*I-HF |
| CAATTG | 6 | 1 | *Mfe*I-HF |
| ACGCGT | 6 | 1 | *Mlu*I-HF |
| CCATGG | 6 | 1 | *Nco*I-HF |
| CATATG | 6 | 2 | *Nde*I-HF |
| GCTAGC | 6 | 1 | *Nhe*I-HF |
| GCGGCCGC | 8 | 2 | *Not*I-HF |
| CTGCAG | 6 | 5 | *Pst*I-HF |
| CAGCTG | 6 | 3 | *Pvu*II-HF |
| GAGCTC | 6 | 5 | *Sac*I-HF |
| CCGCGG | 6 | 4 | *Sac*II |
| GTCGAC | 6 | 1 | *Sal*I-HF |
| TCTAGA | 6 | 1 | *Xba*I |
| CTCGAG | 6 | 1 | *Xho*I |
| CCCGGG | 6 | 3 | *Sma*I |

**Supplementary Table 2.** Necessary API endpoints to access, build, and save custom plasmid project data under an established user account.

| **API Endpoint** | **Request Type** | **Function** |
| --- | --- | --- |
| search_inventory_by_name/ | POST | Searches inventory of plasmids in the service for parts by their annotated name (e.g. pTDH3, CYC1) |
| obtain_all_parts/ | POST | Sends all annotated parts and sequences in inventory of plasmids |
| search_backbone/ | POST | Sends all available backbones from inventory of plasmids after user submits constraints (e.g. organism, GFP included, etc.) |
| create_order/ | POST | Called when user starts a new project to add an order to the database |
| update order/ | PATCH | Called when user makes any edit to their custom plasmid project build and edits the order in the database |
| update_order_name/ | POST | Called when user submits project name change from “Project #” to custom choice |
| get_order/ | POST | Called when user loads an existing project sending all pertinent information of their custom plasmid build |
| submit_order/ | POST | Project cannot be edited and a Celery background task to produce guides, primers, and verification restriction enzymes available to technicians |
| delete_order/ | POST | Removes an order from the database |
| get_order_guides_and_primers/ | POST | Sending a zip file of guides, primers, and restriction enzymes from a particular project |
| get_user_information/ | POST | Sends technician status and projects under user account if user is authorized with a valid authentication token |

**Supplementary Table 3.** The comprehensive information of all the constructed 101 plasmids. Source data are provided as a Source Data file.

| **Species and Plasmid name** | | **Plasmid size** | **Number of correct colonies/total colonies checked** | **Number of fragments** | **Fragment size range** | **Quality control enzyme used** |
| --- | --- | --- | --- | --- | --- | --- |
| *E. coli* | EC_1 | 9017 bp | 2/4 | 5 | 1033-2715 bp | *Eco*RV *& Apa*I |
|  | EC_2 | 9628 bp | 4/4 | 6 | 892-2696 bp |  |
|  | EC_3 | 10507 bp | 1/4 | 6 | 888-2708 bp |  |
|  | EC_4 | 10396 bp | 1/4 | 6 | 889-2706 bp |  |
|  | EC_5 | 10051 bp | 1/4 | 6 | 882-2715 bp |  |
|  | EC_6 | 8594 bp | 4/4 | 5 | 1026-2715 bp |  |
|  | EC_7 | 9473 bp | 4/4 | 5 | 1067-2715 bp |  |
|  | EC_8 | 9362 bp | 4/4 | 5 | 1067-2715 bp |  |
|  | EC_9 | 8791 bp | 2/4 | 5 | 889-2707 bp |  |
|  | EC_10 | 8713 bp | 1/4 | 5 | 876-2700 bp |  |
|  | EC_11 | 10081 bp | 3/4 | 6 | 1066-2707 bp |  |
|  | EC_12 | 10426 bp | 1/4 | 6 | 1066-2707 bp |  |
|  | EC_13 | 10537 bp | 3/4 | 6 | 1066-2707 bp |  |
|  | EC_14 | 8987 bp | 2/4 | 5 | 1147-2714 bp |  |
|  | EC_15 | 8987 bp | 2/4 | 5 | 1147-2714 bp |  |
|  | EC_16 | 9332 bp | 1/4 | 5 | 1142-2705 bp |  |
|  | EC_17 | 9443 bp | 3/4 | 5 | 1146-2715 bp |  |
|  | EC_18 | 8767 bp | 1/4 | 5 | 1066-2707 bp |  |
|  | EC_19 | 7727 bp | 1/4 | 4 | 1033-2715 bp |  |
|  | EC_20 | 7673 bp | 3/4 | 4 | 1146-2715 bp |  |
|  | EC_21 | 6054 bp | 1/4 | 3 | 1041-2706 bp |  |
|  | EC_22 | 6081 bp | 4/4 | 3 | 1056-2715 bp |  |
| *S. cerevisiae* | SC_1 | 9365 bp | 3/4 | 8 | 225-2289 bp | *Bam*HI |
|  | SC_2 | 9385 bp | 2/4 |  | 240-2292 bp |  |
|  | SC_3 | 9385 bp | 2/4 |  | 240-2282 bp |  |
|  | SC_4 | 9385 bp | 3/4 |  | 247-2297 bp |  |
|  | SC_5 | 9384 bp | 1/4 |  | 240-2292 bp |  |
|  | SC_6 | 9385 bp | 2/4 |  | 240-2290 bp |  |
|  | SC_7 | 9405 bp | 4/4 |  | 227-2287 bp |  |
|  | SC_8 | 9405 bp | 4/4 |  | 240-2279 bp |  |
|  | SC_9 | 9405 bp | 2/4 |  | 242-2290 bp |  |
|  | SC_10 | 9404 bp | 3/4 |  | 240-2291 bp |  |
|  | SC_11 | 9385 bp | 2/4 |  | 241-2270 bp |  |
|  | SC_12 | 9405 bp | 2/4 |  | 241-2270 bp |  |
|  | SC_13 | 9405 bp | 2/4 |  | 225-2286 bp |  |
|  | SC_14 | 9405 bp | 1/4 |  | 241-2283 bp |  |
|  | SC_15 | 9404 bp | 3/4 |  | 241-2269 bp |  |
|  | SC_16 | 9385 bp | 2/4 |  | 240-2288 bp |  |
|  | SC_17 | 9405 bp | 2/4 |  | 240-2293 bp |  |
|  | SC_18 | 9405 bp | 4/4 |  | 240-2293 bp |  |
|  | SC_19 | 9405 bp | 4/4 |  | 227-2276 bp |  |
|  | SC_20 | 9404 bp | 1/4 |  | 240-2293 bp |  |
|  | SC_21 | 9384 bp | 3/4 |  | 240-2295 bp |  |
|  | SC_22 | 9404 bp | 4/4 |  | 240-2295 bp |  |
|  | SC_23 | 9404 bp | 3/4 |  | 240-2282 bp |  |
|  | SC_24 | 9404 bp | 2/4 |  | 242-2295 bp |  |
|  | SC_25 | 9404 bp | 3/4 |  | 227-2286 bp |  |
| *I. orientalis* | IO_1 | 11754 bp | 2/8 | 7 | 1009-2743 bp | *Eco*RI *& Pst*I |
|  | IO_2 | 12023 bp | 1/4 | 7 | 619-2742 bp |  |
|  | IO_3 | 12023 bp | 2/8 | 6 | 1000-2743 bp |  |
|  | IO_4 | 11909 bp | 1/4 | 7 | 624-2767 bp |  |
|  | IO_5 | 11909 bp | 2/10 | 5 | 1793-2644 bp |  |
|  | IO_6 | 11640 bp | 3/8 | 7 | 955-2763 bp |  |
|  | IO_7 | 11609 bp | 2/8 | 5 | 1805-2766 bp |  |
|  | IO_8 | 11609 bp | 2/4 | 6 | 623-2738 bp |  |
|  | IO_9 | 11340 bp | 1/8 | 5 | 1810-2646 bp |  |
|  | IO_10 | 12023 bp | 2/4 | 7 | 997-2763 bp |  |
|  | IO_11 | 12023 bp | 3/10 | 6 | 628-2762 bp |  |
|  | IO_12 | 11754 bp | 5/10 | 6 | 918-2763 bp |  |
|  | IO_13 | 11754 bp | 2/8 | 5 | 1777-2644 bp |  |
|  | IO_14 | 11754 bp | 1/4 | 5 | 1796-2654 bp |  |
|  | IO_15 | 11340 bp | 1/8 | 6 | 1006-2738 bp |  |
|  | IO_16 | 11485 bp | 1/10 | 5 | 1790-2649 bp |  |
|  | IO_17 | 11609 bp | 1/8 | 6 | 923-2738 bp |  |
|  | IO_18 | 11609 bp | 2/4 | 6 | 977-2738 bp |  |
|  | IO_19 | 11495 bp | 3/8 | 6 | 517-2738 bp |  |
|  | IO_20 | 11664 bp | 4/4 | 10 | 389-1813 bp |  |
|  | IO_21 | 11664 bp | 4/4 | 8 | 566-1813 bp |  |
| *A. thaliana* | AT_1 | 11902 bp | 4/15 | 5 | 1126-3108 bp | *Sac*II *& Xho*I |
|  | AT_2 | 14023 bp | 3/15 | 8 | 293-3104 bp |  |
|  | AT_3 | 14599 bp | 2/15 | 8 | 302-3104 bp |  |
|  | AT_4 | 14157 bp | 3/15 | 8 | 316-3109 bp |  |
|  | AT_5 | 15096 bp | 3/15 | 8 | 316-3098 bp |  |
|  | AT_6 | 14061 bp | 2/15 | 8 | 313-3103 bp |  |
|  | AT_7 | 15000 bp | 4/15 | 8 | 316-3098 bp |  |
|  | AT_8 | 14865 bp | 2/15 | 8 | 316-3109 bp |  |
|  | AT_9 | 18137 bp | 2/15 | 11 | 310-3099 bp |  |
|  | AT_10 | 17518 bp | 2/15 | 9 | 867-3092 bp |  |
| *HEK 293T* | HEK_1 | 5423 bp | 3/4 | 6 | 493-1215 bp | *Bam*HI *& Eco*RI |
|  | HEK_2 | 5711 bp | 2/4 | 6 | 637-1215 bp |  |
|  | HEK_3 | 5551 bp | 4/4 | 6 | 557-1215 bp |  |
|  | HEK_4 | 7711 bp | 3/4 | 6 | 1088-1662 bp |  |
|  | HEK_5 | 9045 bp | 4/4 | 6 | 1089-2337 bp |  |
|  | HEK_6 | 7203 bp | 4/4 | 6 | 1088-1409 bp |  |
|  | HEK_7 | 7493 bp | 3/4 | 5 | 1000-2335 bp |  |
|  | HEK_8 | 5507 bp | 4/4 | 5 | 519-2335 bp |  |
|  | HEK_9 | 5449 bp | 4/4 | 5 | 490-2370 bp |  |
|  | HEK_10 | 5567 bp | 4/4 | 4 | 770-2335 bp |  |
|  | HEK_11 | 5519 bp | 3/4 | 5 | 542-2328 bp |  |
|  | HEK_12 | 6975 bp | 4/4 | 5 | 765-2328 bp |  |
|  | HEK_13 | 5943 bp | 4/4 | 5 | 754-2328 bp |  |
|  | HEK_14 | 6019 bp | 3/4 | 5 | 765-2328 bp |  |
|  | HEK_15 | 6081 bp | 3/4 | 5 | 765-2328 bp |  |
|  | HEK_16 | 5879 bp | 4/4 | 5 | 722-2328 bp |  |
|  | HEK_17 | 5819 bp | 2/4 | 5 | 692-2328 bp |  |
|  | HEK_18 | 5877 bp | 4/4 | 5 | 726-2323 bp |  |
|  | HEK_19 | 7683 bp | 1/4 | 8 | 399-2329 bp |  |
| *S. griseoflavus* | HighGC_1 | 13142 bp | 6/8 | 5 | 1962-3053 bp | *Eco*RI *& Not*I |
|  | HighGC_2 | 13142 bp | 5/8 | 7 | 1733-2245 bp |  |
|  | HighGC_3 | 13142 bp | 4/8 | 7 | 1449-2059 bp |  |
|  | HighGC_4 | 13142 bp | 4/8 | 8 | 920-2114 bp |  |

# Supplementary Data

**Supplementary Data 1.** DNA sequences of 101 plasmids constructed in this study.

**Supplementary Data 2.** Primers and guide DNA sequences used for construction of 101 plasmids.

# Supplementary References

1. Tong, J., Cao, W. & Barany, F. Biochemical properties of a high fidelity DNA ligase from Thermus species AK16D. *Nucleic Acids Research* **27**, 788-794 (1999).

2. Luo, J., Bergstrom, D.E. & Barany, F. Improving the fidelity of Thermus thermophilus DNA ligase. *Nucleic Acids Research* **24**, 3071-3078 (1996).

3. Lohman, G.J. et al. A high-throughput assay for the comprehensive profiling of DNA ligase fidelity. *Nucleic Acids Research* **44**, e14-e14 (2016).

4. Barany, F. Genetic disease detection and DNA amplification using cloned thermostable ligase. *Proceedings of the National Academy of Sciences U.S.A.*  **88**, 189-193 (1991).

5. Untergasser, A. et al. Primer3—new capabilities and interfaces. *Nucleic Acids Research* **40**, e115-e115 (2012).
