# Supplementary material for: PlasmidMaker is a versatile, automated, and high throughput end-to-end platform for plasmid construction
Source: Nat Commun. 2022 May 16;13:2697. doi: 10.1038/s41467-022-30355-y (PMC9110713; doi:10.1038/s41467-022-30355-y)
Supplement: Supplementary file 2 — Reporting Summary [file 41467_2022_30355_MOESM2_ESM.pdf]

nature portfolio

Corresponding author(s): Huimin Zhao

Last updated by author(s): 4/17/2022

Reporting Summary

Nature Portfolio wishes to improve the reproducibility of the work that we publish. This form provides structure for consistency and transparency in reporting. For further information on Nature Portfolio policies, see our Editorial Policies and the Editorial Policy Checklist.

Statistics

For all statistical analyses, confirm that the following items are present in the figure legend, table legend, main text, or Methods section.

n/a	Confirmed	
☐	☒	The exact sample size (*n*) for each experimental group/condition, given as a discrete number and unit of measurement
☐	☒	A statement on whether measurements were taken from distinct samples or whether the same sample was measured repeatedly
☒	☐	The statistical test(s) used AND whether they are one- or two-sided<br>*Only common tests should be described solely by name; describe more complex techniques in the Methods section.*
☒	☐	A description of all covariates tested
☒	☐	A description of any assumptions or corrections, such as tests of normality and adjustment for multiple comparisons
☐	☒	A full description of the statistical parameters including central tendency (e.g. means) or other basic estimates (e.g. regression coefficient) AND variation (e.g. standard deviation) or associated estimates of uncertainty (e.g. confidence intervals)
☒	☐	For null hypothesis testing, the test statistic (e.g. *F*, *t*, *r*) with confidence intervals, effect sizes, degrees of freedom and *P* value noted<br>*Give P values as exact values whenever suitable.*
☒	☐	For Bayesian analysis, information on the choice of priors and Markov chain Monte Carlo settings
☒	☐	For hierarchical and complex designs, identification of the appropriate level for tests and full reporting of outcomes
☒	☐	Estimates of effect sizes (e.g. Cohen's *d*, Pearson's *r*), indicating how they were calculated

Our web collection on statistics for biologists contains articles on many of the points above.

Software and code

Policy information about availability of computer code

Data collection	Primer3 (version 0.6.0) was used to obtain the Tm for designing the primers. SnapGene-reader (version 0.1.19) was used to read all the 101 annotated plasmids assembled in the manuscript. Momentum (trademark) Workflow Scheduling Software (version 6.0.2) was used to design and build all the processes in automated DNA assembly workflow. An in-house python script was developed to generate guides, primers for plasmid assembly and also to obtain the list of restriction enzymes for assembly verification.
Data analysis	DNA sequences were analyzed by SnapGene viewer software (version 5). Agarose gel band intensities were measured by the imageJ (version 1.53a) software. DNA band sizes for construction of 101 plasmids were analyzed by ProSize data analysis software (version 3.1.0.12)

For manuscripts utilizing custom algorithms or software that are central to the research but not yet described in published literature, software must be made available to editors and reviewers. We strongly encourage code deposition in a community repository (e.g. GitHub). See the Nature Portfolio guidelines for submitting code & software for further information.

Data

Policy information about availability of data

All manuscripts must include a data availability statement. This statement should provide the following information, where applicable:

- Accession codes, unique identifiers, or web links for publicly available datasets
- A description of any restrictions on data availability
- For clinical datasets or third party data, please ensure that the statement adheres to our policy

The data supporting the findings of this study are available within the article and its Supplementary Information files or uploaded through public repositories. If specific data is believed to be missing, that data is available from the corresponding author upon request. The plasmids for expression of PfAgo enzyme and its mutant version will be available through Addgene.

The custom codes for the frontend and backend software used in this study are available at Zenodo: https://zenodo.org/record/5812313 (DOI: 10.5281/zenodo.5812313). The code to design guides, primers for PfAgo-based plasmid assembly and selecting restriction enzymes for assembly verification on a local machine is available at https://github.com/ibiofab/PlasmidMaker_GuideDNA. The primer and guide design tool is also available on the following web address: https://biofoundry.web.illinois.edu/. The datasets used to populate the PostgreSQL database are available in the Supplementary Information files in a SnapGene format. The sequences can be converted into a Postgres format using the database scripts found in our code repository.

Field-specific reporting

Please select the one below that is the best fit for your research. If you are not sure, read the appropriate sections before making your selection.

- [x] Life sciences
- [ ] Behavioural & social sciences
- [ ] Ecological, evolutionary & environmental sciences

For a reference copy of the document with all sections, see nature.com/documents/nr-reporting-summary-flat.pdf

Life sciences study design

All studies must disclose on these points even when the disclosure is negative.

Sample size	No statistical methods were used to predetermine sample size. All method development assemblies were performed with at least three biological replicates. For characterization of DNA sequence requirements for PfAgo/AREs, each experiment was performed only once. These sample sizes were chosen due to time and budget considerations. The results obtained suggest the chosen sample sizes were appropriate because clear distinctions can be seen with the current sample size.
Data exclusions	No data was excluded from analysis. All the experimental/analysis results are included in the manuscript or Supplementary Information.
Replication	All method development assemblies were repeated with three biological replicates. All attempts at replication were successful. For characterization of DNA sequence requirements for PfAgo/AREs, each experiment was performed only once mainly because of time and budget considerations and also because the experiment was designed to see whether the assembly works with moderate to high efficiencies and not to measure the exact efficiency for a specific DNA sequence. The automated construction of 101 plasmids were performed once, but the troubleshooting for obtaining correct PCR amplification products were performed more than three times for some PCRs.
Randomization	No randomization was performed as none of the experiments involved allocation of samples to test groups.
Blinding	Not relevant because no group allocation was involved in this study.

Reporting for specific materials, systems and methods

We require information from authors about some types of materials, experimental systems and methods used in many studies. Here, indicate whether each material, system or method listed is relevant to your study. If you are not sure if a list item applies to your research, read the appropriate section before selecting a response.

Materials & experimental systems

n/a	Involved in the study
☒	☐ Antibodies
☒	☐ Eukaryotic cell lines
☒	☐ Palaeontology and archaeology
☒	☐ Animals and other organisms
☒	☐ Human research participants
☒	☐ Clinical data
☒	☐ Dual use research of concern

Methods

n/a	Involved in the study
☒	☐ ChIP-seq
☒	☐ Flow cytometry
☒	☐ MRI-based neuroimaging
