# Supplementary material for: PlasmidMaker is a versatile, automated, and high throughput end-to-end platform for plasmid construction
Source: Nat Commun. 2022 May 16;13:2697. doi: 10.1038/s41467-022-30355-y (PMC9110713; doi:10.1038/s41467-022-30355-y)
Supplement: Supplementary file 4 — Description of Additional Supplementary Files [file 41467_2022_30355_MOESM4_ESM.pdf]

Title: Supplementary Data 1.
Description: DNA sequences of 101 plasmids constructed in this study.

Title: Supplementary Data 2.
Description: Primers and guide DNA sequences used for construction of 101 plasmids.
